# Some Points in Invalid Feeding

**Published:** 1903-05-16

**Authors:** 


### SOME POINTS IN INVALID FEEDING.

There is no doubt that many patients die because they do not get sufficient nourishment, and for this reason a reminder of some of the less frequently employed methods of administering food to the sick may not be out of place. Whenever it is possible the various foods and methods of feeding by mouth should be tried. Small quantities of albumen water, made by beating up the whites of three eggs in a pint of cold boiled water with an added pinch of salt, given frequently are rapidly absorbed. Beef tea, with the addition of gelatine or isinglass, barley water, milk and lime water, whey or raw meat-juice mixed with a little cream, are less likely to be vomited than ordinary milk. For a child a nourishing concoction is made by boiling chopped suet wrapped in a muslin bag in fresh milk. It is unnecessary to speak of the better-known foods and proprietary preparations, although many of them are most satisfactory nutrients. If the patient is vomiting everything, a blister to the epigastrium will occasionally arrest the sickness. In the case of an unconscious patient the stomach or nasal tube should be resorted to without unnecessary delay. A pint of peptonised milk three times in 24 hours is usually enough for an adult.

Rectal feeding is extensively used in gastric ulcer and other "medical" diseases of the stomach, but neglected far too much by the surgeon. Where, as not infrequently happens after severe abdominal operations, the patient cannot keep the smallest quantity of food in the stomach, a pint of peptonised milk is easily retained when given per rectum. The technique of administering large nutrient enemata is simple and very important. The rectum must be washed out with 2 or 3 pints of soap and water, and then after half an hour's rest a rectal tube, connected with a douche can, is passed up as high as possible. The level of the fluid in the can must not be higher than 1 foot above the rectum of the patient. A satisfactory nutrient mixture to be used in this way consists of unboiled peptonised milk 1 pint, glycerine of carbolic acid 10 minims, and common salt one teaspoonful. This fluid takes not less than three-quarters of an hour to run in, the patient often sleeping the whole time. If the rectum be very irritable two grains of powdered opium suspended in starch mucilage poured into the tube before the milk will act as a sedative. Albumen water with 5 per cent. dextrose may be used instead of milk, and is readily absorbed. Lauder Brunton recommends a wash out once in 24 hours consisting of 3 pints of thin Benger, of which part is retained and absorbed. A smaller quantity of nourishment given per rectum in the above way is valuable in the treatment of wasting children. Olive oil enemata given with the pelvis raised are often partially absorbed, and patients have grown obese in consequence of their frequent use. Many attempts have been made to administer nourishment by means of semi-solid digested food introduced into the rectum, but only with slight success. It might be expected that the rectum, naturally containing semi-solid material, would retain this better than milk, but it does not appear that this is the case.

Sometimes a patient cannot be fed by mouth or rectum; in such a case certain forms of nourishment may be introduced subcutaneously. This method is simple and devoid of risk if the following procedure is carefully adhered to:—A solution is prepared containing ʒx of best French honey or ℥i pure dextrose to one pint of saline solution. This is placed in a large flask plugged with burnt brown wool and boiled for 20 minutes, then strained by pouring through a funnel plugged lightly with burnt wool, into a douche can. The funnel is left in place during the subsequent procedures so that it may keep out dust. A tube from the douche can is connected with a sharp cannula. All this apparatus must be boiled before use in a large fish kettle or similar steriliser. The skin close to one breast, preferably the right, is cleansed and a drop of pure carbolic painted on with a match, the trocar is inserted beneath the breast and a pint of the fluid, at a temperature of from 105°–115° Fahr., allowed to run in rather slowly; this may be repeated every four or six hours, using alternate breasts, axillæ or loins. To prevent the pain caused by the insertion of trocar two drops of 1 per cent. cocaine may be injected just beneath the epidermis, *i.e.* into, not beneath, the skin. Horse serum, isinglass, or gelatine may be used as more or less satisfactory substitutes for dextrose, but are more difficult to sterilise.

Lastly, in the case of wasting children and emaciated adults fat can be introduced through the unbroken skin. For this purpose cod-liver oil, olive oil, or ordinary lard is rubbed into the skin twice or three times a day. Among the methods of ancient physicians was the bath containing meat essence, veal broth, or goat's milk; it is uncertain whether in any case this nutriment was absorbed at all, but there is no doubt that fat can be successfully used as a nutrient inunction.

